# Simulation of thalamic prosthetic vision: reading accuracy, speed, and acuity in sighted humans

**DOI:** 10.3389/fnhum.2014.00816

**Published:** 2014-11-04

**Authors:** Milena Vurro, Anne Marie Crowell, John S. Pezaris

**Affiliations:** Department of Neurosurgery, Massachusetts General Hospital/Harvard Medical SchoolBoston, MA, USA

**Keywords:** visual prosthesis, artificial sight, neuroprosthesis, neurotechnology

## Abstract

The psychophysics of reading with artificial sight has received increasing attention as visual prostheses are becoming a real possibility to restore useful function to the blind through the coarse, pseudo-pixelized vision they generate. Studies to date have focused on simulating retinal and cortical prostheses; here we extend that work to report on thalamic designs. This study examined the reading performance of normally sighted human subjects using a simulation of three thalamic visual prostheses that varied in phosphene count, to help understand the level of functional ability afforded by thalamic designs in a task of daily living. Reading accuracy, reading speed, and reading acuity of 20 subjects were measured as a function of letter size, using a task based on the MNREAD chart. Results showed that fluid reading was feasible with appropriate combinations of letter size and phosphene count, and performance degraded smoothly as font size was decreased, with an approximate doubling of phosphene count resulting in an increase of 0.2 logMAR in acuity. Results here were consistent with previous results from our laboratory. Results were also consistent with those from the literature, despite using naive subjects who were not trained on the simulator, in contrast to other reports.

## Introduction

Restoring sight to the blind is a challenge that researchers around the globe have been addressing through a variety of approaches, from genetics (e.g., Acland et al., 2001; Beltran et al., 2012), to replacement surgery (e.g., corneal transplant or keratoprosthesis as in Zerbe et al., 2006) to visual prostheses (reviewed in Mertz, 2012; Ong and da Cruz, 2012) such as the device-based approach our laboratory has been investigating (Pezaris and Reid, 2007, 2009; Pezaris and Eskandar, 2009; Bourkiza et al., 2013; Jeffries et al., 2014). Electrically-based visual prostheses operate on the principle that current passed through an electrode implanted in the early stages of the visual pathway (retina, optic nerve, thalamus or primary visual cortex) evokes the sensation of a spot of light or *phosphene*. An appropriate set of electrodes, the field thus hypothesizes, could be used essentially as a direct-to-brain display to evoke a more complex visual scene through patterned stimulation, allowing researchers and ultimately physicians to bypass the damaged structures and provide restoration of function. Among such devices, the thalamic visual prostheses proposed by our group have the potential to restore high resolution vision and be applied to a wide variety of causes of blindness, from retinal disease, to cancer, to trauma (Pezaris and Reid, 2007).

Recent reviews have discussed the relative merits of different approaches to device-based artificial vision (e.g., Zhou and Greenberg, 2009; Dagnelie, 2011), including the thalamic approach (Pezaris and Eskandar, 2009) we have been pursuing. The LGN, in particular, provides an excellent target, having a well-understood retinotopic map, functional characteristics that closely match the retinal ganglion cell layer, macroscopic segregation of the magno-, parvo-, and konio cellular pathways, and, thanks to the unrelated field of deep brain stimulation (DBS, reviewed in Bronstein et al., 2011), routine surgical access. This last point bears some amplification: DBS had become a common clinical therapy for treatment of movement disorders such as Parkinsonian tremor, providing symptomatic relief through electrical activation of stimulating electrodes placed in structures that are 1–2 cm from the LGN. Given nearly 100,000 patients with DBS implants worldwide (Tierney et al., 2011), safe and reliable surgical access to the mid-brain for stimulating electrodes is, essentially, a solved problem, overcoming the primary barrier to use of the LGN as a target for artificial vision.

The current report continues our efforts to assess the performance of thalamic visual prosthesis designs that intend to implant electrodes in the dorsal lateral geniculate nucleus of the thalamus (Pezaris and Reid, 2009; Bourkiza et al., 2013). In preceding work, we used a simulation of thalamic vision to investigate the relationship between electrode number and visual acuity using a standardized test in sighted humans (Bourkiza et al., 2013). Here, we use a more advanced simulation to examine the same relationship for reading performance, again employing a standardized test in sighted humans. While a few studies have investigated the interaction of device parameters on reading acuity and speed for retinal prosthetic vision (Cha et al., 1992; Humayun, 2001; Hayes et al., 2003; Sommerhalder et al., 2003, 2004; Pérez Fornos et al., 2005, 2011; Dagnelie et al., 2006; Fu et al., 2006), this is the first such study for thalamic prosthetic vision.

Reading is involved in several fundamental tasks both for work and leisure, and is considered an activity of daily living (Dagnelie, 2008). Reading is a more complex ability than letter recognition alone as it requires the integration of several additional cognitive and perceptual processes. A person who is reading must first visually acquire and process the words presented, then match them to stored semantic representations, and finally combine these representations to create a meaningful sentence. To create a standard measurement of reading ability in normal and low-vision subjects, several reading acuity charts (Legge et al., 1989b; Radner et al., 1998) have been developed, of which the Minnesota Reading Acuity (MNREAD) test is one of the most common (Crossland et al., 2008). Consisting of a series of simple three-line sentences, shown in font sizes that decrease proportionally from one sentence to the next, the MNREAD chart is used to measure reading accuracy (the percentage of correctly recognized words), reading acuity (the smallest size of print that the patient can reliably resolve), reading speed (the number of words per minute that are read correctly), and critical print size (the smallest print that a subject can read while maintaining their maximum reading speed).

Although standard tests have been widely used to evaluate reading ability for retinal diseases (Virgili et al., 2004; Cappello et al., 2009; Uppal et al., 2011), glaucoma (Ramulu et al., 2009, 2012; Burton et al., 2012), and aging (e.g., Sass et al., 2006), only three studies on visual prosthesis reading performance have employed a standard reading chart (Humayun, 2001; Hayes et al., 2003; Fu et al., 2006) and only one the MNREAD standard test procedure (Fu et al., 2006), while the rest have used *ad-hoc* methods (Cha et al., 1992; Sommerhalder et al., 2003, 2004; Pérez Fornos et al., 2005, 2011; Dagnelie et al., 2006). This is unfortunate as variations in test methodologies make it difficult to accurately compare results between studies (see Discussion). The effects of experimental and methodological variation are underscored by Legge and colleagues who, in the past three decades, have shown that, for normal and low vision, reading performance is affected by pixel density to character size ratio (Legge et al., 1985a), contrast (Legge et al., 1987; Rubin and Legge, 1989), font type (Mansfield et al., 1996), spacing and size (Legge et al., 1985b, 1997), word size (Legge et al., 1997), drifting or static text (Legge et al., 1989b), cognitive content of the text (Legge et al., 1989a) and central or peripheral vision (Legge et al., 2001). Since the artificial vision studies reported in the literature employed different values for the parameters investigated by Legge, the results for simulated prostheses have been influenced not only by the experimental parameters (e.g., number of simulated electrodes, electrode drop outs, or phosphene spacing and simulation characteristics) but also by differences in methodology, including stimulus design.

In this study, we sought to investigate the reading accuracy, speed and acuity of sighted humans in a simulation of thalamic prosthetic vision. To accomplish this, we constructed a real-time simulation of artificial vision including current understanding of what recipients of a future thalamic prosthesis are likely to experience based on previous work from our laboratory (Pezaris and Reid, 2007, 2009; Pezaris and Eskandar, 2009). As in our previous report on simulated thalamic vision (Bourkiza et al., 2013), we base our experimental task on a standardized method, here the MNREAD test, to simplify cross-laboratory comparisons.

We tested three phosphene pattern densities spanning anticipated device complexity, and six font sizes spanning 1.0–1.5 logMAR. Text shown directly on the screen without filtering, designated as *clear* in the reminder of this paper, was used as a control condition. We hypothesized that reading accuracy for simulated prosthetic vision would be similar to clear-text reading for the largest font size combined with high phosphene counts, and would decrease proportionally with letter size. We further expected reading speed to be in general lower than clear-text reading and to vary with letter size and phosphene count. Finally, we anticipated that comparable results could be obtained to our previous work with isolated letter recognition (Bourkiza et al., 2013) but in much less time and with methods more agreeable to subjects.

## Methods

### Overview

Subjects performed a reading task. Images of the text were manipulated in real-time so as to simulate the perception of a thalamic visual prosthesis wearer. Overall design followed the MNREAD test, including text taken directly from the chart. During testing, two experimental parameters were varied: the viewing condition, either normal viewing as a control or simulated artificial sight using one of three phosphene patterns with approximately 2000, 1000, and 500 electrodes; and the letter size, decreasing from 1.50 to 1.0 logMAR in steps of 0.1 logMAR. By design, participation required a total of about 20 min per subject.

### Subjects

Twenty-four subjects (9 M, 15 F; range 19–50 years of age) participated in the study. Subjects, recruited from students and post-docs at Massachusetts General Hospital (MGH) and the general population, were required to have self-reported normal or corrected-to-normal vision, and be able to read English text. Subjects were assigned pseudonyms for the purpose of anonymizing data collection and received modest monetary compensation for their participation.

### Ethics statement

The research protocol used in this study was approved by the MGH Institutional Review Board and adhered to the guidelines of the Declaration of Helsinki. As this study was classified as a minimal risk experiment, verbal consent was obtained from each subject, and was implied by the existence of a data record.

### Apparatus

**Figure 1:**
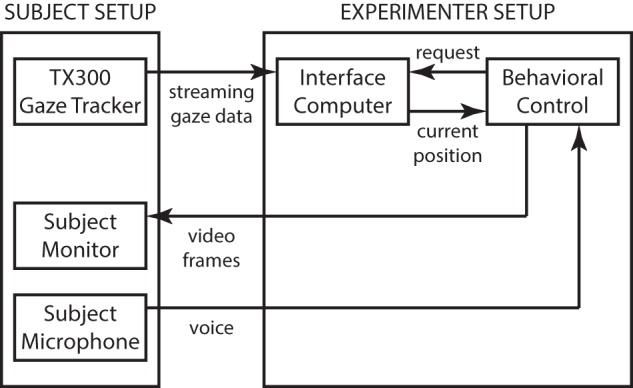
**System architecture**. The system consists of the Tobii TX300 gaze tracker operating in normal, streaming mode. Gaze position information relative to the TX300's built-in display screen is streamed over a low-latency dedicated connection to an intermediary *interface* computer that runs a small gaze-position server program. The server code accepts streaming data and, upon request from the *behavioral control* system, computes an instantaneous gaze position value (with non-linear noise reduction), that is returned over a second, low-latency dedicated connection. The behavioral computer runs the experiment and performs data logging. Whenever the behavioral task requires instantaneous gaze information (such as during the Reading Phase of the task; see main text), a request is sent to the gaze-position server that typically replies in under 2 ms. Total system latency from gaze measurement to video frame update is typically under 16 ms, with an additional 4 ms of LCD lag to an updated image on the subject retina. The intermediary gaze-position server eliminates the need for the behavioral control system to deal with streaming data, and thus simplifies the overall design, at the cost of a modest increase in total system latency.

The experimental apparatus consisted of a heads-free binocular gaze tracker with integrated display (TX300, Tobii, Inc.), and two additional computers (M92p, Lenovo, Inc.) running custom-written software for interfacing, behavioral control, and data collection (Figure 1). The gaze tracker provided streaming gaze information at 300 Hz (0.4° accuracy and 0.14° precision) that was received and processed on the *interface* computer to be made available upon periodic request from the *behavioral control* computer running the experiment. The behavioral control computer coordinated experimental activities, including computing stimuli and presenting them on the TX300 integrated display, and logged experimental data. A small consumer-grade computer microphone was used to record audio during the experiment for *post-hoc* blind verification of subject performance. The stimulus display was operated at the native 1920 × 1080 resolution with 60 Hz vertical refresh rate. With the standard viewing distance of 65 cm, the display subtended 43° × 25° of visual angle.

**Figure 2:**
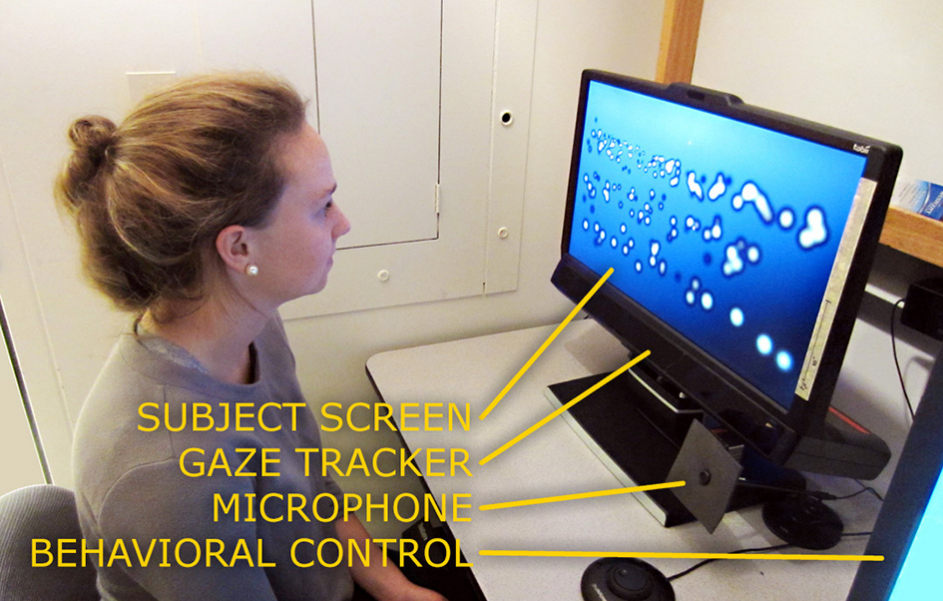
**A subject in front of the apparatus**. A subject is shown seated in front of the Tobii TX300 gaze tracker, in a typical position for performing the experiment. No chin bar or other head stabilization is necessary with the TX300. The stimulus display screen in front of the subject shows a frame from the Reading Phase where a collection of phosphenes can be seen to very roughly depict three lines of large text. The bluish screen background and dark halos around the phosphenes are artifacts from the off-axis viewing angle of the camera; to the subject, the background appears uniformly black and the phosphenes as white Gaussians (see Figure 3).

The apparatus was located in a small office with normal levels of lighting. Subjects were seated in front of the TX300 that was placed on a normal desk-height table (see Figure 2). An office chair without casters was used to prevent subjects from moving too far away from the optimal distance from the screen. The experiment control displays were arranged to one side of the TX300 and positioned such that they did not visually distract the subjects. An experimenter was present during all data collection.

**Figure 3:**
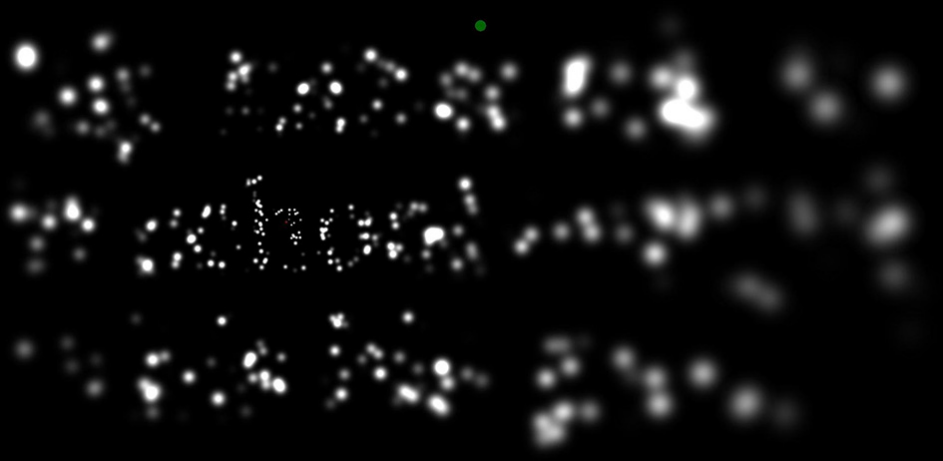
**A screen image taken during the reading phase of the task**. Here, the simulation of thalamic artificial vision with 2000 simulated phosphenes (far fewer are engaged in this image, due to the limited extent of the display, and to the particular bright/dark pattern forming the text) is shown with the subject looking at text displayed at the largest font size. The image depicts the text “My father takes me/to school every day/in his big green car” with the point of regard alighted on the *h* in *school*. The details of image generation for phosphene vision are found in Figure 4. As the point of regard moved around the screen, the pattern of phosphenes shifted accordingly, revealing more detail wherever the subject was looking. Because of the ability of the human visual system to integrate information across eye movements, the text appeared far more legible as a whole than this static image would imply. Every subject was able to read at the depicted condition (largest text with densest phosphene pattern) with relative ease and 100% accuracy.

**Figure 4:**
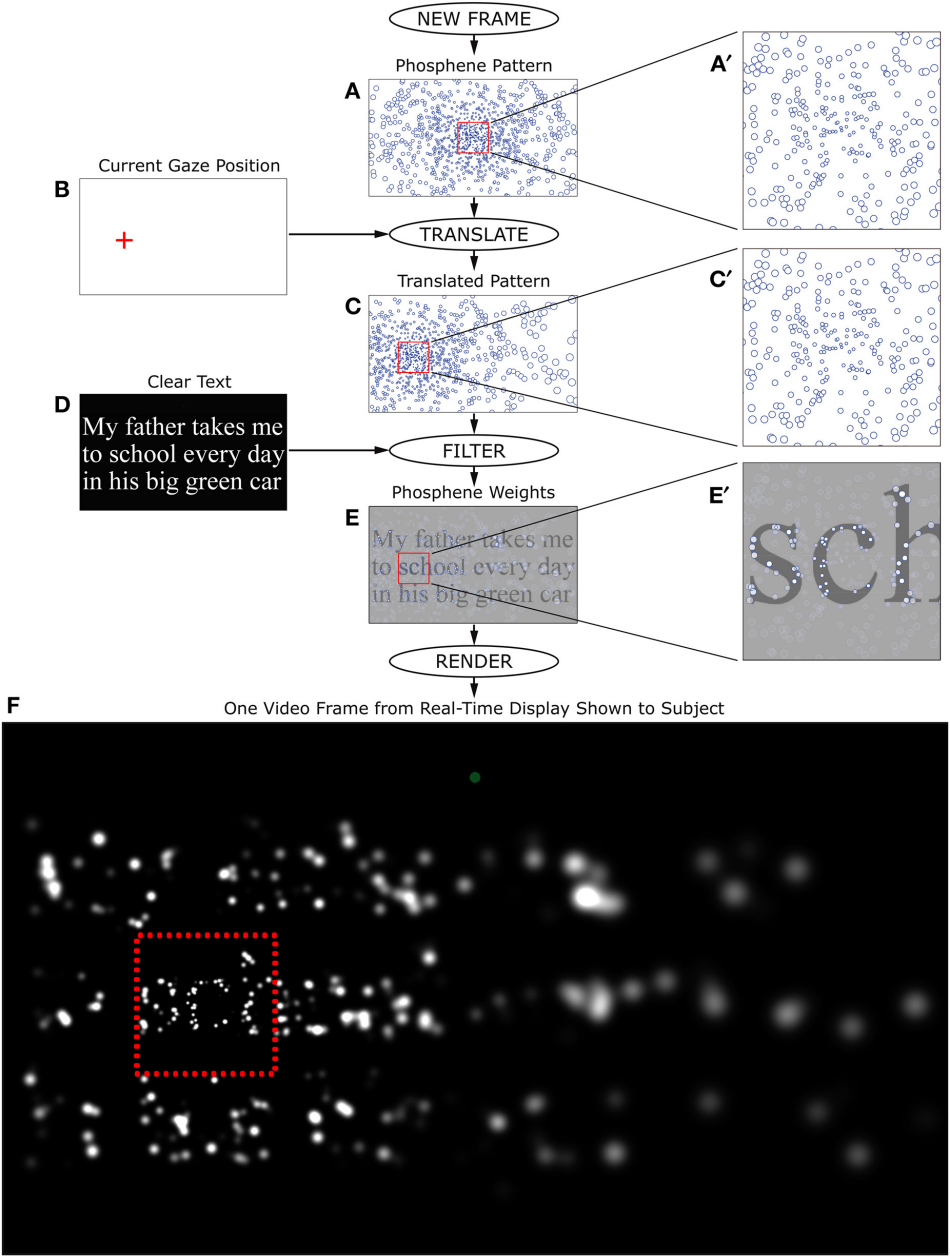
**Real-time stimulus generation**. Each frame during the Reading Phase of phosphene vision trials was generated in real time according to the flow chart shown here. The phosphene pattern selected for the trial **(A)**, was translated in visual space so that the location (0, 0) was centered on the instantaneously read gaze position relative to the screen **(B)**, to generate a pattern relative to the point of regard **(C)**. The text for the given trial was then rendered as an off-screen image **(D)**, and the translated pattern of locations overlaid on that image as a set of independent averaging filters. Each phosphene brightness was determined by taking the local average luminance of the text image weighted by a two-dimensional Gaussian filter that was sized according to the eccentricity of the phosphene (see main text). The outputs of individual filters **(E)** were used to set the brightness of matching-sized Gaussians at the corresponding locations. When phosphenes overlapped, they were combined additively in the final image. After all phosphenes were rendered **(F)**, the entire frame was copied to the video card. Typical processing time for each frame was less than the refresh time of the subject monitor, so that each monitor refresh could contain a new update, and thus create a real-time simulation. Since the phosphene pattern was always translated to the point of regard, the procedure had the effect of (coarsely) stabilizing phosphene locations on the retina, matching the expected behavior of a real device (Pezaris and Reid, 2007). The frame generated here corresponds to the example shown in Figure 3.

Substantial effort was put into optimizing stimulus generation so as to minimize system latency, including careful selection of the video card used for stimulus generation (Asus AMD Radeon HD7750-1GD5-V2, ASUSTeK Computer, Inc.; the HD7750 is not a high-end card, but one that had the best 2-D performance of the dozen or so tested). Typical delays from eye movement to an updated screen image (including monitor screen latency) were 20 ms or less, with a maximum of 37 ms. The longer delays were often seen with the densest phosphene pattern, and rarely, if ever, with the sparser patterns. An image taken from one frame of the screen during the simulation is shown in Figure 3. Details of how the phosphene locations were approximately stabilized on the retina through frame-by-frame gaze-contingent stimulus generation are described in the caption to Figure 4.

### Procedure

At the beginning of the experiment each subject was comfortably seated at a distance of about 65 cm from the stimulus display. Specific instructions were given for the calibration and reading tasks which were then performed in that order.

#### Calibration

The calibration task consisted of a series of small dots that appeared one at a time in a 3-by-3 array of locations spanning the stimulus monitor. Subjects were instructed to, “look as closely and accurately at each dot as possible.” These fixation points were presented in a balanced, interleaved fashion. The first nine presentations (one for each location) were used to trigger TX300 calibration. The subsequent 27 presentations (three for each location) were used to calibrate a second-order non-linear correction in the experimental software that automatically accounted for gain, offset, and minor distortions by fitting a two-dimensional parabaloid to the calibration points (as typically only gain and offset were required, the TX300's output could be used without this additional correction). Upon occasion of a poor TX300 calibration as subjectively assessed by the experimenter (caused, for example, if the subject blinked at an inopportune time), the procedure was repeated.

#### Experiment

**Figure 5:**
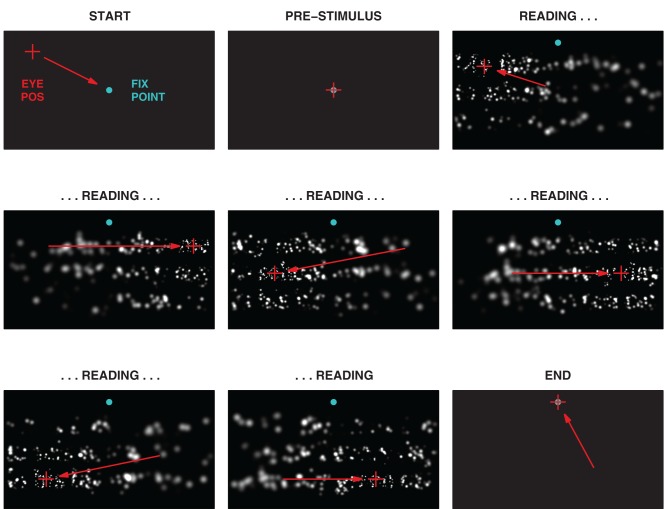
**Phases of the experimental task**. One trial of the task is shown in a sequence of snapshots. The task had four distinct phases, Start, Pre-Stimulus, Reading, and End, with the bulk of the time spent in the Reading phase. Green-blue dots indicate the initial central fixation point used to engage the trial, and the subsequent dot at the center top used to advance to the next trial. Red crosses indicate the instantaneous gaze position, and red arrows, the gaze motion from one snapshot to the next (neither would appear to the subject). During the Reading phase, the subject is free to look about the screen, but gaze patterns typically followed the three lines of text with a series of fixations on each line. The (simulated) subject in this instance can be seen to read across each of the three lines, left to right and top to bottom, before looking to the trigger point in the End phase. The image in the center panel corresponds to the image in Figure 3.

The experiment consisted of a single block of 48 trials. Each trial in the block was subdivided into a series of four phases, *Start, Pre-Stimulus, Reading*, and *End* (see Figure 5). During the Start Phase, a fixation point appeared in the middle of the screen that the subject was required to foveate in order to engage the experiment. Once foveated for the duration of the Pre-Stimulus phase, the fixation point was extinguished. The Reading phase then followed with one of the sentences displayed along with an additional dot near the top center of the screen. The subject was required to read the sentence out loud, as quickly and accurately as possible, or to declare their inability to read it. In order to advance to the next sentence, subjects looked at the top center dot. Subjects could take as long as they wanted, consistent with reading quickly and accurately. Once the subject foveated the top center dot for 350 ms, the trial entered the End Phase, the screen was blanked, and a 2000 ms pause provided an intertrial interval before the next trial commenced. An audio recording was made for the entirety of each experiment.

Each subject was presented an identical sequence of stimuli that varied in experimental condition. Mimicking the presentation used in MNREAD assessment, sentences were shown starting with a large font size and progressing to smaller ones. For each font size, text was presented in four viewing conditions, first in the clear as a control, and then in artificial vision simulation with three sets of phosphene patterns with decreasing phosphene count. The overall progression was therefore from easiest to most difficult in steps. After the full set of combinations was carried out, the conditions were repeated with a fresh set of sentences. Each sentence was presented exactly once, and each combination of viewing condition and font size presented exactly twice for a completed experiment (see Appendix A in Supplementary Material).

#### Snellen screening

To verify approximately normal vision, each subject was administered a standard Snellen chart task. Subjects were instructed to stand at a mark on the floor 20 feet away from the vertical surface where the Snellen chart was affixed. No attempts were made to control lighting beyond making sure the overhead lights were on to provide consistent, ordinary levels of illumination. The task was performed binocularly at a pace determined by the subject. Corrective lenses were worn if the subject normally used them and preferred to do so. Measurements were all expressed in equivalent logMAR units.

### Stimuli

Forty-eight sentences from the MNREAD chart were used to create the experimental stimuli. These sentences are in simple English that is suitable for readers 8 years old or older. Each sentence contains 60 characters including spaces, and from 10 to 13 words. Sentences were displayed across three lines broken as evenly as possible without hyphenation.

Each sentence was rendered in Times New Roman (as available as a system font under Microsoft Windows) with a regular weight and style at one of six font sizes (from 1.5 logMAR down to 1.0 logMAR in steps of 0.1 logMAR, corresponding to the sizes of the lowercase *x*, see Table 1), in white letters on a black background. Depending on the trial conditions, the sentence was presented on the screen in the clear at the native monitor resolution, without any manipulations, or in a simulation of prosthetic vision using one of three phosphene patterns that varied in the number and density of phosphenes (see Table 2) but shared a common overall center-weighted profile based on previous work (Pezaris and Reid, 2009). The full sequence of stimulus conditions, including the text of each sentence, can be found in Appendix A (Supplementary Material). While some researchers studying low-vision reading use sans-serif fonts, we opted to mimic the readily available MNREAD charts (Lighthouse Low Vision Products, Long Island City, NY) for clinical compatibility; these charts use a serif font in the Times Roman class.

**Table 1:** **Font conditions and sizes**.

**Font condition (*f*)**	**Font size (logMAR)**
1	1.5
2	1.4
3	1.3
4	1.2
5	1.1
6	1.0

*The variable f is used in the formulas for reading accuracy α, reading speed β, and reading acuity γ described in the Methods Section. The largest font that allowed the MNREAD sentences to properly fit on the monitor as three lines had an x height of 2.9 cm, corresponding to logMAR 1.5 at the viewing distance of 65 cm*.

**Table 2:** **Viewing conditions and reading acuity**.

**Viewing condition (*v*)**	**Phosphene pattern density**	**Total phosphenes (count)**	**Central (10°) phosphenes (count)**	**Electrode spacing (μm)**
1^*^	n/a	n/a	n/a	n/a
2	High	1757	381	375
3	Medium	1029	231	475
4	Low	522	124	600

The first viewing condition, marked with an asterisk (

* *) is the Clear condition where text is presented without a simulation of artificial vision as a control. The variable v is used in the formulas for reading accuracy α, reading speed β, and reading acuity γ described in the Methods section*.

### Simulated prosthetic thalamic vision

During the Reading Phase of trials where the stimuli were presented in a simulation of prosthetic vision, the text did not appear directly on the screen, but, rather, a virtual filter was placed in front of the text and the output of the filter shown on the screen to give the illusion of prosthetic vision. The simulation is described in detail in a previous publication (Bourkiza et al., 2013), and is summarized as follows. All current artificial prosthesis designs that include stimulation through sets of microwire contacts provide the recipient with a coarse visual experience made up of a set of isolated pixel-like percepts called phosphenes. The size and distribution of the set of phosphenes for a given device is part of the device design, being a function of both electrode pattern and visual field map in the stimulated structure; the thalamic visual prosthesis forming our line of inquiry is expected to elicit a pattern of phosphenes that is denser toward the center of vision, and, for lower electrode counts, is relatively sparse (Pezaris and Reid, 2009; Bourkiza et al., 2013). The pattern of phosphenes is referenced to retinal coordinates (Pezaris and Reid, 2007) and thus moves about with the direction of gaze, as do after-images or retinal features like the blind spot. Our simulation approximated this effect by taking the instantaneous gaze position from the gaze tracker, translating the position of the set of phosphenes to that location on the monitor, and then activating each phosphene according to the brightness of the image of the text at the corresponding location (details in Figure 4). This can be thought of as similar to looking through a colander held at arms length: as the colandar is moved about, different parts of the scene behind are revealed. For initially conceived devices, such as described by Pezaris and Reid (2007), where an external video camera is mounted on a set of glasses worn by the patient, a mechanism will be needed to read the instantaneous eye position and electronically shift the video left or right and up or down, frame by frame, according to the point of regard.

Each phosphene was drawn as a white, circular Gaussian, and was assumed to be independent of all other phosphenes. When phosphenes overlapped, they were combined additively, saturating at the maximum brightness available from the monitor. Phosphene size σ (the one-sigma extent of each Gaussian) varied as a linear function of radial distance ρ from the point of regard (or, eccentricity), according to the formula σ = 0.043 ρ + 0.083 (see Discussion), in degrees of visual angle.

### Experimental parameter: phosphene pattern density

Three different densities of phosphenes were investigated, all following the global density profile expected from a thalamic visual prosthesis (Pezaris and Reid, 2009), and selected for near-future engineering plausibility. The underlying profile is denser toward the point of regard, and reflects the endogenous acuity profile across the visual scene in monkey LGN. The three sampling densities of the profile, *High, Medium*, and *Low*, corresponded to devices with approximately 2000, 1000, and 500 phosphenes spanning the entire visual field (see Figure 2 for an example rendering). The exact phosphene counts, along with the number of phosphenes in central vision, can be seen in Table 2.

### Analysis

Three aspects of subject ability in the reading task were analyzed: reading accuracy (the percentage of correctly recognized words), reading speed (the number of correctly recognized words per minute, or WPM), and reading acuity (the subject's visual acuity assessed through reading). Our analysis formulae are generalizations of the published MNREAD formulas (Mansfield et al., 1994), with extensions for the number of font sizes, the step from one font size to the next, and the number of repeated observations. In conditions matching those of a traditional MNREAD task, the expressions reduce exactly to published formulae. The extensions described provide additional mathematical insight along with the flexibility to support a broader range of conditions with precision, so are presented in detail as a reference for the field.

#### Reading accuracy

The ability to accurately recognize words is a fundamental parameter reflecting available visual utility. The corresponding quantification of reading accuracy, α, is computed for each trial *class* (the combination of font *f* and viewing condition *v*) as the mean of the *N* observations of the normalized number of words read correctly in each sentence. For the present experimental design that contains exactly two trials for each (*f, v*) combination, *N* is 2. As the standard in the field is to record the number of words missed when scoring performance, α is computed using the number of words in each sentence, *n*, minus the number of words read incorrectly or not read at all, *e*, pooled over observations *i* by trial class (*f, v*):

(1) $$\alpha_{f\text{​},v}=\frac{1}{N}\sum_{i}\frac{n_{f\text{​},v,i}-e_{f\text{​},v,i}}{n_{f\text{​},v,i}}.$$

#### Reading speed

The speed with which words are recognized and vocalized is also a fundamental parameter reflecting available visual utility. The corresponding quantified value of reading speed, β, is computed as the mean of the number of words correctly read in each sentence divided by the time *t* it took a subject to read them, again pooled over observations *i* by trial class (*f, v*):

(2) $$\beta_{f\text{​},v}=\frac{1}{N}\sum_{i}\frac{n_{f\text{​},v,i}-e_{f\text{​},v,i}}{t_{f\text{​},v,i}}.$$

The value of *t* for each trial was defined as the time spent in the Reading Phase, and did not include the 350 ms required to activate stepping to the next trial.

#### Reading acuity

Subject performance was used to derive a visual acuity measurement γ, following the standard methods for the MNREAD task. The sum of reading accuracies across font sizes was interpreted as the fractional number of size intervals down from the largest font that the subject was effectively reading. As the data have already been pooled over observations, reading acuity is computed by pooling over font sizes *f* as the base font size, Δ_0_ (here 1.5 logMAR), plus the sum of accuracies weighted by the incremental difference in size from one font size to the next, Δ, giving us a result for each viewing condition *v:*

(3) $$\gamma_{v}=\Delta_{0}+\sum_{f}\Delta_{f}\alpha_{f\text{​},v}.$$

Note that values of Δ after Δ_0_ are negative because the font sizes are ordered largest to smallest, and that for the general case of uneven font size steps, the values of Δ_*f*_ will not be uniform. To verify the values of γ with a more robust method, reading acuity was also determined by the midpoint of logistic fits to the population data (using asymptote values of 0 and 100%). Logistic fitting is considerably more robust, as it, importantly, has better noise rejection, in addition to allowing for uneven intervals, missing observations, and sampling that stops short of spanning the entire transition band.

Each of the values for reading accuracy α, reading speed β, and reading acuity γ, were calculated for each subject, and subsequently combined into population values to be shown in the figures presented below. Statistical tests were applied as described in the Results Section to assess significance at the *p* < 0.05 level.

## Results

### Subjects

During the Snellen chart procedure preceding the main experiment, no subjects were excluded for poor visual acuity, or other obvious visual issues. The population average visual acuity was found to be 0.09 ± 0.16 logMAR (mean ± *SD*), with a range of –0.24 to 0.29 logMAR, using per-letter scoring methods (Ferris et al., 1982).

During the experiment, however, four subjects were unable to complete the session or had problems reading the text on the screen. One subject reported double vision as a normal (for the subject) condition unrelated to the experiment. One subject could not read the simulation text without extreme difficulty at even the largest size. Finally, for two subjects, we were unable to obtain reliable eye-tracking. These four subjects were considered outliers and their results have been excluded from additional analysis, as well as from the Snellen acuity reported above.

In the first trial with the simulation engaged (the second overall trial, see Appendix A in Supplementary Material), despite being an easy condition, subjects often exhibited unusually slow reading speed because of a one-time latency as they took a moment to understand the simulation. This would have been eliminated with a practice round, but including a practice round would have introduced a deviation from the MNREAD standard task that was initially considered unacceptable (see Discussion for potential improvements to task design). Excluding the first simulation trial for each subject from our data analysis does not materially alter the presented results.

As a population, subjects were 66.3 ± 3.0 cm (mean ± *SD*) away from the display, quite close to the ideal of 65 cm. Each subject's standard deviation from their own mean position was, on average, 1.4 cm. The minimum per-subject mean position was 61.0 cm, and the maximum was 74.5 cm. The maximum occurred with a subject with unusually large pupils, intentionally placed somewhat farther away from the screen in order to obtain reasonable tracking. Excluding the minimum and maximum values results in little change to the population figure at 66.0 ± 2.1 cm. Mean per-subject positions appeared to have a normal distribution (Kolmogorov–Smirnov test, *p* = 0.8). The simulation did not compensate for different subject positions, as that would have potentially resulted in the largest text being clipped by the screen. Data below are reported assuming all subjects were seated at the standard distance of 65 cm (see Discussion).

### Reading accuracy

**Figure 6:**
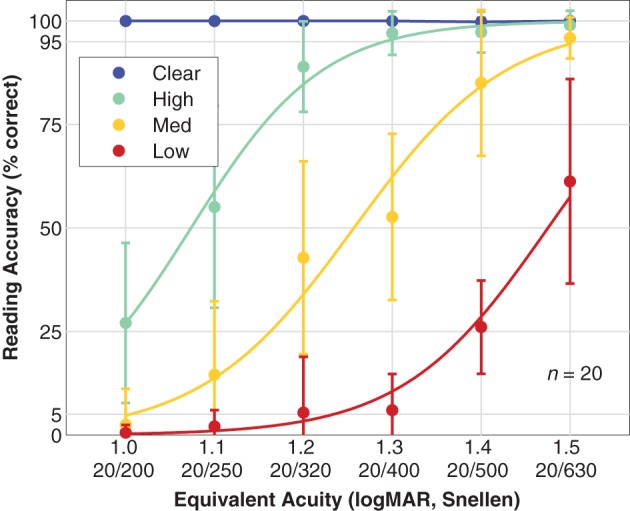
**Reading accuracy**. Population reading accuracy vs. font size, expressed as equivalent acuity is shown. Data have been grouped by viewing condition: Clear (blue; text presented in the clear as a control condition), High density phosphene pattern (green), Medium density pattern (yellow), and Low density pattern (red). Data points are the mean, error bars are the standard deviation of population values. Smooth curves are logistic fits with assumed asymptotes of 0 and 100% for all three phosphene vision conditions.

Figure 6 shows population reading accuracy α as a function of font size pooled by viewing condition. Subjects were able to accurately read all of the control sentences as expected since the clear texts were all shown with font sizes substantially larger than the subjects' visual acuity as measured with the Snellen chart. For simulated prosthetic vision, the population performances form a family of three curves shifted to varying degrees depending on phosphene count. For the High density phosphene pattern, the first observation below 95% accuracy for the population mean is at 1.2 logMAR, and the 50% performance threshold from the fitted logistic curve is at 1.07 ± 0.07 logMAR (mean ± *SD*, slope of 340%/logMAR). For the Medium density phosphene pattern, we see a more complete logistic curve with the first observation below 95% at 1.4 logMAR (although the 1.5 logMAR value at 96% is very close), and the 50% level at 1.26 ± 0.06 logMAR (slope of 290%/logMAR). If the pattern continues, as we might expect from the matching shapes of the three curves, then the Low density phosphene pattern would first drop below 95% at 1.6 logMAR, as is supported by the 50% level at 1.48 ± 0.09 logMAR (slope 310%/logMAR), a nearly equal distance from the Medium pattern 50% level as the Medium is from the High.

### Reading speed

**Figure 7:**
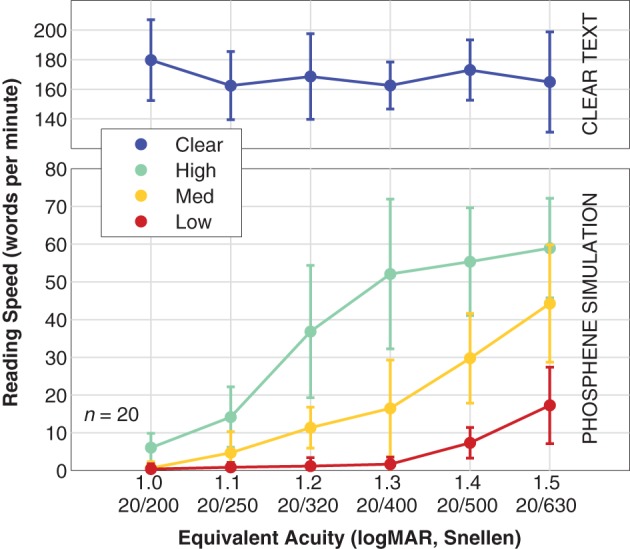
**Reading speed**. Population data are shown for reading speed vs. font size (expressed as equivalent acuity), broken down by viewing condition. The **upper panel** shows the Clear condition where text is shown in the clear as a control (blue). The **lower panel** shows the results for simulated artificial vision with the three phosphene patterns, High density (green), Medium density (yellow), and Low density (red). Points are the mean, error bars are the standard deviation of population values. The two panels have different vertical scales; the control viewing condition has been separated out to display more detail in the simulation viewing conditions. Logistic fits were not performed with these data as only one condition spanned lower to upper performance plateaus as necessary for a valid fit, and we do not have direct evidence that the asymptotes are identical for all three conditions. As at least two curves would be necessary for meaningful comparisons, a fit for the High data is not shown.

Reading speed β was faster for text presented in the clear than for text presented in artificial vision simulation, and varied in a pattern similar to reading acuity (see Figure 7). For the control viewing condition, population reading speed was independent of stimulus condition, as the letters were well above the critical font size, although a slight elevation in speed appears to happen for the smaller end of the size scale (we speculate that this might be due to fewer or smaller head motions necessary to read the text). For the simulation conditions, reading speed varied from essentially 0 WPM (0.40 ± 1.4 WPM, mean ± *SD*) for the most difficult combination (smallest font size at 1.0 logMAR equivalent, and Low density phosphene pattern) to a maximum of 59 ± 13 WPM for the easiest combination (1.5 logMAR font size with High density pattern). With the High density pattern, the transition from full-speed reading down to no reading has been captured, but we see only parts of the transition for the Medium and Low density patterns. Logistic fits were not made to these data as there is no *a priori* upper asymptote that can be used for the Medium and Low data.

### Reading acuity

Population reading acuity γ as a function of viewing condition for both the direct fixed-interval method described above, as well as the midpoint value of the logistic fits to accuracy data are shown in Table 3. Acuity measurements were limited by the range of font sizes used, and thus the reported acuity is 1.00 ± 0.00 logMAR (mean ± *SD*) for text shown in the clear; this value corresponds to the smallest font tested because the actual value is out of range. Reading acuity for phosphene patterns was: 1.14 ± 0.05, 1.31 ± 0.06, and 1.50 ± 0.03 logMAR (mean ± *SD*) for High, Medium, and Low density patterns, respectively, as computed with the MNREAD-based formula for γ from Equation 3. When computed as the midpoint of logistic fits to the reading accuracy data, as reported above, the values for reading acuity are somewhat lower at, 1.07 ± 0.07, 1.26 ± 0.06, and 1.48 ± 0.09 logMAR (mean ± *SD*), respectively. These data show that an approximate doubling of phosphenes in the patterns from one step to the next results in an improvement in acuity of about 0.2 logMAR.

**Table 3:** **Reading acuity**.

**Viewing condition (*v*)**	**Phosphene pattern density**	**MNREAD acuity (logMAR)**	**Logistic acuity (logMAR)**	**Logistic slope (%/logMAR)**
1^*^	n/a	1.00 ± 0.00	n/a	n/a
2	High	1.14 ± 0.05	1.07 ± 0.07	338
3	Medium	1.31 ± 0.06	1.26 ± 0.06	292
4	Low	1.50 ± 0.03	1.48 ± 0.09	305

The first viewing condition, marked with an asterisk (

* ) is the Clear condition where text is presented in the clear as a control, without a simulation of artificial vision, as presented in Table 2. The values for reading acuity computed with the MNREAD technique, are shown for each phosphene pattern, illuminating the relationship between the number of phosphenes and the performance of the simulated artificial vision provided: Increasing the number of phosphenes by an approximate factor of 2 results in an improvement of about 0.2 logMAR in acuity. Acuity for the control condition was limited by the set of font sizes used, and is a very loose upper bound (contrast with the population mean acuity of 0.09 logMAR from the Snellen chart task). Values are population mean and standard deviation of per-subject computed values. MNREAD and logistic-fit acuities for each phosphene viewing condition were not significantly different from normal distributions (Kolmogorov–Smirnov test, *p* > 0.5 in all cases). The logistic acuity values represent the 50% threshold value (see Figure 6), a more robust metric than the MNREAD computation γ, and are consistently lower than the MNREAD values.

**Figure 8:**
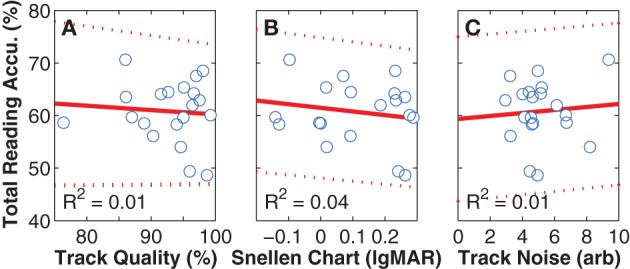
**Instrumentation effects**. The panels in this figure depict per-subject mean values (blue unfilled circles), along with least-squares linear regression fits (red solid lines) and 95% confidence intervals (red dashed lines). **(A)** *Reading Accuracy vs. Tracking Quality*. With an *R*^2^-value of close to zero, a relationship was not found between reading accuracy α_*total*_ (the per-subject mean over all viewing conditions and font sizes) and tracking accuracy (the percentage of valid 300 Hz samples). Removing the outlier with 76% tracking does not materially affect the result. Tracking quality can be less than 100% because either of blinks, or loss of localization of the eyes by the gaze tracker. In both cases, the gaze-position sever code provided values to the simulation that were gracefully degraded by either interpolating across missing values or repeating the last good value. **(B)** *Reading Accuracy vs. Snellen Chart Acuity*. With an *R*^2^-value also close to zero, a relationship also was not found between performance on the simulated vision task and the visual acuity of normal sight as measured on the Snellen chart task, although a trend was seen where better natural acuity lead to slightly better performance on the task. Again, removing the datum at 76% tracking as an outlier does not affect the outcome. **(C)** *Reading Accuracy vs. Tracking Noise*. With an *R*^2^-value again close to zero, a relationship was not found between performance on the simulated vision task and noise in the tracking signal. When the same three comparisons were performed for reading speed instead of accuracy, the *R*^2^-values were slightly higher, with 0.02, 0.04, and 0.06, respectively, but still close to zero. When performed for reading acuity, they were to 0.01, 0.04, and 0.01, respectively. The combination of these analyses suggest that we did not have instrumentation problems that would have led to unintended bias in the primary results.

As all subjects had better visual acuities in the Snellen chart assessment than the smallest font size used in our experiment, any interaction between the acuity of normal vision and task performance would indicate either an instrumentation or a simulation problem. Fortunately, no such relationship was found (Figure 8A). Similarly, we looked for and did not find correlations between reading accuracy and either tracking quality, or noise in the reported gaze position (Figures 8B,C), suggesting that there were no inherent problems in instrumentation.

## Discussion

### Comparison with previous work from our laboratory

In a previous study we investigated the effect of phosphene count on visual acuity in an isolated letter recognition task with a simulated thalamic visual prosthesis (Bourkiza et al., 2013). The phosphene layouts used in that study were highly similar to the ones used in the present report, save for total phosphene count. The most reliable way of comparing the two results is to count the number of phosphenes contained within a circle corresponding to five times the size of the measured acuity, matching to the five dark/light bands across the vertical aspect of the smallest reliably identifiable letter E. This measurement gives a fundamental metric of *perceptual efficiency*. In our previous report, this value was found to be a relatively constant 20 ± 2 phosphenes (mean ± *SD*), and was argued to be consistent with the necessity of sampling two-dimensional patterns in the recognition task. In the present report, the numbers are similar at 21 ± 3, and the null hypothesis that the two distributions are the same is not disproven (paired *t*-test, *p* = 0.8). We unexpectedly did not see an improvement due to the contextual effects of reading full text in the present work rather than isolated letters in the previous work.

A substantial improvement was seen, however, with subject fatigue and length of participation. In our previous work using the isolated letter task, subjects were required to perform nearly 2000 trials, typically over two 1-h sessions with frequent breaks. In comparison, subjects in the current study were typically finished in 20–25 min without any breaks, and did not report any of the drowsiness seen previously.

### Comparisons with previous work from other laboratories

Although there is an existing literature of reading performance studies on simulated retinal prostheses, this is the first study concerned with reading performance on a simulated thalamic prosthesis. Comparison with previous studies from other laboratories can therefore be somewhat difficult. We will first review studies for which a direct comparison is not possible, and then those for which it is. A condensed summary of the literature is presented in Appendix B (Supplementary Material).

The ranges of tested parameters or methodological approach do not match up, for example, with work by Cha et al. (1992), Humayun (2001), or Hayes et al. (2003). Cha and colleagues were the first to examine reading speed in prosthetic vision, simulating phosphenes as regularly spaced holes in a physical mask with subjects reading text from a popular book or reading assessment tests. Their least dense pattern had 100 electrodes in a uniform grid spanning a 1.7° square. Using letters 0.7 logMAR in size, they reported about 50 WPM reading speed with a gaze-contingent setup (see their Figure 3). Using letters at 1.0 logMAR, they reported 75 WPM for an automatic scrolling setup (see their Figure 2). For the same window our High density pattern has only 45 electrodes, below half of the count in their pattern, and the smallest letters we tested were at 1.0 logMAR; that combination had nearly no effective reading (Figure 7). The second study, by Humayun, reported 7 of 8 subjects were able to read text at the equivalent of logMAR 1.5 at 18 WPM with an array of 16 × 16 pixels, but scanning the text by hand in front of a fixed camera feeding a head-mounted display; they also reported one subject experienced motion sickness with the setup. At the same font size, our Medium pattern has a comparable number of phosphenes in central vision (231), and we report 45 WPM (Figure 7); the substantial difference is likely due in part to the inherent slowness of manually scanning a printed card in front of the camera in Humayun's study. The last of these three studies (Hayes et al., 2003) reported that with 64 simulated phosphenes in a 11.3° × 19.3° area, subjects had a maximum reading speed of 18 WPM with letters that were 1.7 logMAR, and a reading acuity of 1.5 logMAR (although at a speed of 7 WPM). Our Low resolution pattern provided the same reading speed at 1.5 logMAR, and an extrapolated reading acuity of 1.5 logMAR, but given the limited overlap in the tested font sizes and the mismatch in phosphene count, the comparison carries little weight.

When other studies were comparable with the present one, we observed comparable performance levels, despite not having trained our subjects unlike every other study. Sommerhalder et al. (2003) showed that subjects were able to read at 110 WPM with 90% reading accuracy at 1.6 logMAR with an array of 286 pixels in a visual area of 10° × 3.5°. For the same area our High resolution pattern has three-quarters the density with 218 electrodes, and we obtained 59 WPM/99% accuracy at 1.5 logMAR (our largest tested). Sommerhalder and a slightly different set of colleagues (2004) reported three highly-trained subjects reading at a mean of 94 WPM at 1.3 logMAR with an array of 572 pixels in a visual area of 10° × 7°. For the same area our High resolution pattern has about 60% the density with 352 electrodes, and we obtained 52 WPM/97% accuracy at the same size of 1.3 logMAR. Pérez Fornos et al. (2005, 2011) showed 60 WPM with over 90% reading accuracy at 1.3 logMAR with 572 pixels in a viewing window of 10° × 7°. We obtained very similar performance with our High resolution pattern that has 352 phosphenes (60% as many) in the same area. Dagnelie et al. (2006) explored the effects of grid scale, number of gray levels, and contrast for four subjects, using a scale-agnostic method, reporting a best-case speed of 52 WPM with a 25 × 25 square array spanning 4.5 characters in width (see their Figure 4). Our best-case speed is slightly better at 59 WPM using the High density pattern with 1.5 logMAR letters, 4.5 of which span about 9.6°, representing about 410 electrodes (65% as many). Finally, Fu et al. (2006) examined the performance for a series of phosphene densities in a fixed window of 5.7° × 5.7°, testing reading speed for a range of letter sizes. For the most comparable conditions to the present study, they measured maximum reading speeds of 32, 46, and 58 WPM that occurred at 1.5, 1.4, and 1.3 logMAR for 64, 100, and 225 phosphene arrays, respectively. Our Low, Medium, and High density arrays yielded 17, 30, 52 WPM at the same font sizes with 73, 134, and 223 phosphenes in the same visual area, respectively, however for none of our patterns were these the maximal reading speeds.

Substantial methodological differences exist beyond the quantified differences above, some of which are summarized in Appendix B (Supplementary Material). The most important differences concern the subjects: Many studies employed a small number of subjects (six or fewer) and required subjects to undergo several days to weeks of training. In contrast, we used a larger cohort who were naive to artificial vision, were not familiar with the MNREAD task, and performed no training prior to data collection. Thus, our results should be seen in light of these differences and we speculate that with systematic training subject reading accuracy and speed would both improve substantially. Supporting that speculation, we find that the authors' performance on the present task (excluded from the presented data) to be strikingly better than that of the naive participants.

### Scoring methods

The standard MNREAD scoring strategy, as described in the Methods Section, can be expressed in a compact, simple form that is easy to calculate. However, it lacks robustness under non-ideal circumstances, such as if the testing range does not include the entire transition from full-speed reading to not reading at all, if the font sizes are not regularly spaced either by design or if a condition is inadvertently skipped during test administration, or if there is a non-trivial false negative rate, especially before the transition is encountered. Fitting a logistic function to the data provides robustness against these cases. When we compared the logistic fits to the MNREAD scoring, we found that the logistic results were consistently lower (the reported acuity was better), as shown in Table 3. Additional testing is required to provide proper validation.

### Acuity vs. phosphene count

The results in Table 3 demonstrate that an approximate doubling of phosphene count resulted in an increase of about 0.2 logMAR acuity. Given that doubling the number of phosphenes across the two-dimensional visual field would result in an expected increase in only 0.15 logMAR acuity, the results bear closer consideration. The precise electrode counts that were used are only an approximate doubling. When the actual phosphene numbers are taken into account (see Table 2), we see that from High to Medium patterns, there is an expected improvement of 0.15 logMAR, and from Medium to Low, 0.12 logMAR. Comparing against the observed increases of 0.22 and 0.19 logMAR, respectively, we see that there is a consistent surplus of 0.07 logMAR. That is, for the parameter range tested, subjects were able to integrate more information than expected from a simple sampling density argument. This surplus requires additional investigation.

### Subject viewing distance

Actual subject distance varied slightly from the reference distance of 65 cm, as reported above. The measured population mean position (66.3 cm) was about 1 cm farther away from the screen, which would have the effect of improving all reported acuities slightly. We elected to ignore the variation in this report for the sake of simplicity, as the expected impact of the population mean distance vs. the standard distance would be about 0.01 logMAR. This prediction was verified by re-computing the logistic fit reading accuracy with font sizes adjusted for each individual's distance, and the change was an improvement of acuity of 0.01, 0.02, and 0.02 logMAR for High, Medium, and Low patterns, respectively, from the values reported in Table 3. As described below, future work is anticipated to automatically compensate for instantaneous viewing distance to improve the test.

### Quality of gaze tracking

Despite using infrared light to track gaze position, the TX300 requires relatively high levels of visible illumination in order to drive subject pupils small enough for reliable localization. Of our participants, the TX300 had some level of trouble for eight of them (below 95% tracking quality in Figure 8A), and two additional subjects had to be disqualified due to tracking issues as described above. As the intent is to simulate artificial vision as accurately as possible, the ideal setup would have the subject sitting in the near-dark with the only luminance source being phosphenes on the stimulus screen, or a featureless gray environment with the screen background matching the room luminance. However, despite the unexpectedly wide range in tracking quality, we found no interaction between tracking and subject reading performance, suggesting that it had limited effect on the overall experiment (Figures 8A,C).

### Considerations on phosphene patterns

Our work, along with perhaps all of the reports from the literature, assumes knowledge of the placement of each phosphene in the visual field to a high degree of accuracy. For implants with more than a handful of electrodes in any part of the visual pathway, determining this mapping is a large challenge that, as yet, has no definitive solution, and remains a critical issue for future clinical work. In animal models where normal sight exists, a mapping of phosphene location can be made from the response fields of cells at the tip of each electrode (Pezaris and Reid, 2007). In individuals without functional visual input, such as in a blind patient receiving an implant, other methods must be used. A map based on anatomical placement of electrodes in the stimulated area may prove an acceptable, if crude, starting point to be refined through recipient feedback, but an automated method needs to be developed. An interesting variant of the present work would be to mimic such inaccuracy by intentionally distorting the map so that phosphenes represent luminance from slightly dislocated parts of the visual field.

The maps resulting from a related distortion that introduced a systematic radial displacement would be equivalent to adding a lens in front of the camera to either expand or compress the visual scene. Measuring visual acuity under such conditions presents an additional challenge because of the confounding effects of the lens. With normally functioning eyes which have an integral non-zoom lens, acuity measurements represent an assessment of the functioning of the entire apparatus of sight. We speculate that a different measurement will be necessary for artificial visual systems, and hope that the idea of perceptual efficiency (Bourkiza et al., 2013) described above will prove useful to the field.

It should be noted that there may also be long-term modification of the perceived location and size of each phosphene in visual space due to plasticity at both thalamic and cortical levels, similar to retinal rewiring known to occur in some degenerative retinal diseases (e.g., Marc and Jones, 2003). It is not, at present, known what sort of rewiring may be taking place in the LGN with disease-driven loss of sight, other than general atrophy (Gupta et al., 2009). We speculate that prosthesis phosphene maps may therefore need periodic updating, in addition to the initial individual specialization mentioned above, to accommodate such changes.

### Gaze contingency

The simulation as described in this work involves a gaze-contingent presentation to approximately stabilize the phosphenes on the retina. As mentioned above, the class of prostheses represented must perforce contain eye-tracking components; miniature eye trackers are currently available in goggle or eye-wear mounts from multiple manufacturers (Tobii Technology, Sweden; SensoMotoric Instruments, Germany). It is difficult to speculate how the present results would be impacted for a head-only gaze contingent system, other than the likelihood of reading speeds being lower, as head movements are typically slower than eye movements (e.g., Freedman and Sparks, 1997).

### MNREAD sentences and learning

The number of published, validated MNREAD sentences is limited to a relatively small set and represents one of the factors constraining the design of this experiment. This constraint prevents, for example, a careful examination of learning effects through a higher number of repeats per condition, and accordingly longer experience with the simulation. Although some work has been done to expand the corpus with an automated generator (Crossland et al., 2008), an expanded, validated set would be a boon to researchers in the field.

### Future directions

There are ways in which we will seek to make improvements for future work, including revisions to test design, simulation accuracy, and apparatus as described in the next paragraphs.

Adjustments to the experimental design will be necessary to make more accurate measurements. As we expect training effects from the new visual modality depicted in the simulation, interleaved stimulus conditions are necessary to eliminate the potential bias from the standard MNREAD method that presents text in order of decreasing font size. Similarly, although the MNREAD task calls for one or two repeats, additional repetitions would decrease test–retest uncertainty, and improve statistical robustness. A larger or wider stimulus screen would allow the presentation of larger fonts, and enable capturing more of the sigmoid curves for lower-density phosphene patterns; this observation is particularly important given that contemporary and near-future devices have relatively few electrodes and are thus expected to produce relatively few phosphenes. Finally, given previous evidence for training effects from our laboratory (Bourkiza et al., 2013) and others (Sommerhalder et al., 2003; Dagnelie et al., 2006), it would be highly informative to perform a longitudinal study to elucidate adaptation to phosphene vision, rather than studying purely naive subjects such as used here. Studies on naive subjects are, however, important as they set a baseline for what can be expected immediately post-implant for potential future human work when recipients are also, initially, naive.

Improvements in the knowledge of human LGN can be used to improve the accuracy of our simulation. The retinotopic mappings that are available (Uğurbil et al., 1999; Kastner et al., 2004; Schneider et al., 2004) regrettably do not meet the requirements for this study, either in completeness of the visual field, or in anatomical detail. Moreover, although we have good evidence that the size of phosphenes is related to the receptive fields at the tip of the electrode in monkeys (Tehovnik and Slocum, 2007; Schiller et al., 2011), we do not yet have well-controlled confirmation from microstimulation in humans (although see Brindley and Lewin, 1968). We do know that receptive field sizes vary in rough proportion with the magnification factor, as a function of eccentricity, and work by Cowey and Rolls (1974, see especially their Figure 3) allows us to use acuity as a stand-in for magnification factor. Visual acuity in humans has been well-studied, and we used data from Anderson et al. (1991, see especially their Figure 5), depicting visual acuity in cycles per degree across the full horizontal meridian. We found the distribution of the inverse of acuity across eccentricity could be well-approximated by a linear expression that was in turn adapted to a scale of phosphene size. Confirmation was provided by the some reports from the literature of phosphene size at various eccentricities (Humayun et al., 1996, 2003; Schmidt et al., 1996; Rizzo et al., 2003), including unpublished reports from our laboratory which indicate phosphenes from microstimulation of monkey LGN are approximately 0.5° across at 10° eccentricity.

Finally the simulation itself could be improved through advances in technology. Specifically, further reductions in latency are desirable, through, for example, monitors with faster refresh rates, and more powerful computers. The display in the TX300 suffers from off-axis color shifting that a higher quality monitor would correct. Given that there are individual variations in acuity—and presumably therefore variations in phosphene size from hypothetical visual pathway microstimulation—a more accurate simulation would also adjust the phosphene size distribution to each participant. With a larger subject display, the simulation could also compensate for distance variations from subject to subject (or even moment to moment for a given subject) to ensure that stimuli were perceived at the intended logMAR sizes. Finally, a reduction in eye tracking instrumentation uncertainty to more fully stabilize the phosphene locations on the retina would be highly desirable through, for example, more advanced noise reduction algorithms.

## Conclusion

This presentation embodies the first quantification of reading performance with a simulated thalamic visual prosthesis, with results demonstrating that reading using a real thalamic device should be of at least comparable performance to reading with a retina prosthesis, with the potential for substantial improvement through training. The simulation used for this work extends our previous efforts in isolated letter recognition to an activity of daily living with more realistic utility. Our findings show that equivalent per-electrode performance is reached in both tasks, with the present central result being an improvement of 0.2 logMAR in acuity for doublings of phosphene count with the three patterns tested. Importantly, the method used here is based on a standardized model and is easily and rigorously comparable with results from laboratories that take similar approaches.

We speculate that the addition of training, through longer exposure to the simulation, and improvements in simulation design, through more accurate physiological models and higher performance hardware, could improve subject ability on the task. The exact characteristics of the electrophysiological response of the lateral geniculate nucleus to patterned electrical stimulation remain undetermined to this date. A substantial research effort is therefore still needed to solve these and other open issues to create a more accurate representation of the perception of a thalamic visual prosthesis wearer. We hope that the strengths and adaptability of our simulation will allow it to be used in the future to quickly assess device parameters and directly compare different approaches.

### Conflict of interest statement

The authors declare that the research was conducted in the absence of any commercial or financial relationships that could be construed as a potential conflict of interest.
