# Supplementary material for: Simulation of thalamic prosthetic vision: reading accuracy, speed, and acuity in sighted humans
Source: Front Hum Neurosci. 2014 Nov 4;8:816. doi: 10.3389/fnhum.2014.00816 (PMC4219440; doi:10.3389/fnhum.2014.00816)
Supplement: Supplementary file 1 [file DataSheet1.PDF]

Supplementary Material

Simulation of thalamic prosthetic vision: reading accuracy, speed, and acuity in sighted humans

Milena Vurro, Anne Marie Crowell and John S. Pezaris*

Department of Neurosurgery, Massachusetts General Hospital/Harvard Medical School, Boston, MA, USA

*** Correspondence:** John S. Pezaris, Department of Neurosurgery, Massachusetts General Hospital, 55 Fruit Street, M/C THR-425, Boston, MA, USA e-mail: pezaris.john@mgh.harvard.edu

APPENDIX A: Stimulus Conditions and Sentences

Seq	*Size*	*Viewing*	*Text*
1	1.5	Clear	My father takes me | to school every day | in his big green car
2		High	Everyone wanted to | go outside when the | rain finally stopped
3		Medium	They were not able | to finish playing the | game before dinner
4		Low	My father asked me | to help the two men | carry the box inside
5	1.4	Clear	Three of my friends | had never been to a | circus before today
6		High	My grandfather has | a large garden with | fruit and vegetables
7		Medium	He told a long story | about ducks before | his son went to bed
8		Low	My mother loves to | hear the young girls | sing in the morning
9	1.3	Clear	The young boy held | his hand high to ask | questions in school
10		High	My brother wanted | a glass of milk with | his cake after lunch
11		Medium	I do not understand | why we must leave | so early for the play
12		Low	It is more than four | hundred miles from | my home to the city
13	1.2	Clear	Our father wants us | to wash the clothes | before he gets back
14		High	They would love to | see you during your | visit here this week
15		Medium	The teacher showed | the children how to | draw pretty pictures
16		Low	Nothing could ever | be better than a hot | fire to warm you up
17	1.1	Clear	The old man caught | a fish here when he | went out in his boat
18		High	Our mother tells us | that we should wear | heavy coats outside
19		Medium	One of my brothers | went with his friend | to climb a mountain
20		Low	The three elephants | in the circus walked | around very slowly
21	1.0	Clear	We could not guess | what was inside the | big box on the table
22		High	The two friends did | not know what time | the play would start
23		Medium	She wanted to show | us the new toys she | got for her birthday
24		Low	The mother told her | son that she wanted | him to go to school
25	1.5	Clear	An old man took a | picture of my sister | and her little puppy
26		High	Ten different kinds | of flowers grow by | the side of the road
27		Medium	Put your first name | on this paper if you | will help tomorrow
28		Low	The father gave his | children some fruit | for lunch every day
29	1.4	Clear	Please do not make | noise while they are | reading their books
30		High	We sometimes take | long walks together | if it is warm outside
31		Medium	I must always clean | my room before the | football game starts
32		Low	My little sister went | swimming each day | during our vacation
33	1.3	Clear	The children in my | school want to have | their lunch outdoors
34		High	One of the students | brought a big apple | for the new teacher
35		Medium	The wind lifted our | red kite high above | the huge maple tree
36		Low	The little boy ate | so much candy that | he soon grew very big
37	1.2	Clear	There is a big river | to cross before you | reach the mountain
38		High	He liked playing in | the school band but | could not play well
39		Medium	He never could sing | any of the songs we | played on the piano
40		Low	There are two dogs | and three cats in the | park near my house
41	1.1	Clear	The snow fell softly | this morning before | our family woke up
42		High	Many people came | to help us clean the | place after the party
43		Medium	He could see a bird | outside if he looked | through his window
44		Low	The teacher wanted | the children to learn | how to draw a boat
45	1.0	Clear	We like to listen to | music when we are | eating our breakfast
46		High	Three of my closest | friends are going to | visit him tomorrow
47		Medium	She gave a glass of | water to her mother | before going to bed
48		Low	My brother was not | feeling very well so | he did not go today

APPENDIX B: Summary of Results from the Literature.

Basic parameters from experiments published in the literature of reading performance with simulations of artificial vision devices are shown, along with values from the present report.

Report	*Phosphene simulation type*	*Number of simulated phosphenes*	*Viewing window (WxH, in degrees)*	*Standardized text*	*Other parameters varied*	*Training prior to experiment*
Cha et al. (1992)	Perforated opaque mask (isolated pixels modulated in brightness, black background)	100<br>256<br>625<br>1024	Square:<br>0.54 x 0.54<br>to<br>1.7 x 1.7	No:<br>4-8 grade texts	Scanning: Single page or scrolling	Yes
Humayun (2001)	Unformly gray dots in rectangular grid, black backround	16<br>60<br>256	Rect:<br>7.3 x 7.3<br>11.3 x 19.3<br>11.3 x 11.3	Yes:<br>MNREAD	Printed cards manually scanned in front of fixed position camera	Yes
Hayes et al. (2003)	Square uniformly gray continuous pixels varying in brightness, black background	60<br>256	Rect:<br>11.3 x 19.3	Yes:<br>MNREAD	Gray-scale levels	Yes
	Gaussian profile pixels, isolated, 8 gray levels, black background	60			Varying drop-out, phoshene size, electrode spacing	
Sommerhalder et al. (2003)	Square uniformly gray continuous pixels varying in brightness, white background	83<br>140<br>286<br>875	Rect:<br>20 x 7<br>10 x 3.5	No:<br>French<br>4-letter words	Eccentricity, monocular / binocular presentation	Yes
Sommerhalder et al. (2004)	Square uniformly gray continuous pixels varying in brightness, white background	572	Rect:<br>10 x 7	No:<br>French newspaper articles	Eccentricity: foveal / peripheral	Yes
Pérez Fornos et al. (2005)	Square uniformly gray continuous pixels or Gaussian profile pixels, isolated, *n* gray levels, gray background	166<br>280<br>572<br>1750<br>28000	Rect:<br>10 x 7	No:<br>French newspaper articles	Real time or off-line presentation (monocular)	Yes
Fu et al. (2006)	Isolated white dots, black background	36<br>64<br>100<br>225<br>400<br>1024	Square:<br>5.7 x 5.7<br>13.7 x 13.7	Yes:<br>MNREAD	Window width, phosphene size, monocular / binocular presentation	Yes
Dagnelie et al (2006)	Isolated gray dots, black background	100<br>256<br>625	Rect:<br>36 x 48	No:<br>6th grade text	Dot size and spacing, dropout, number of gray levels	Yes
Current experiment	Isolated Gaussians, center-weighted pattern, varying brightness, black background (see text)	522<br>1029<br>1757	Rect:<br>43 x 25<br>(see text)	Yes:<br>MNREAD	None	No
